# Supplementary figures and images for: Comparative Analysis of Muscle Metabolome and Amino Acid Profiles in All-Female Rainbow Trout (Oncorhynchus mykiss) from Different Germplasm Sources
Source: Biology (Basel). 2025 Nov 18;14(11):1613. doi: 10.3390/biology14111613 (PMC12650459; doi:10.3390/biology14111613)

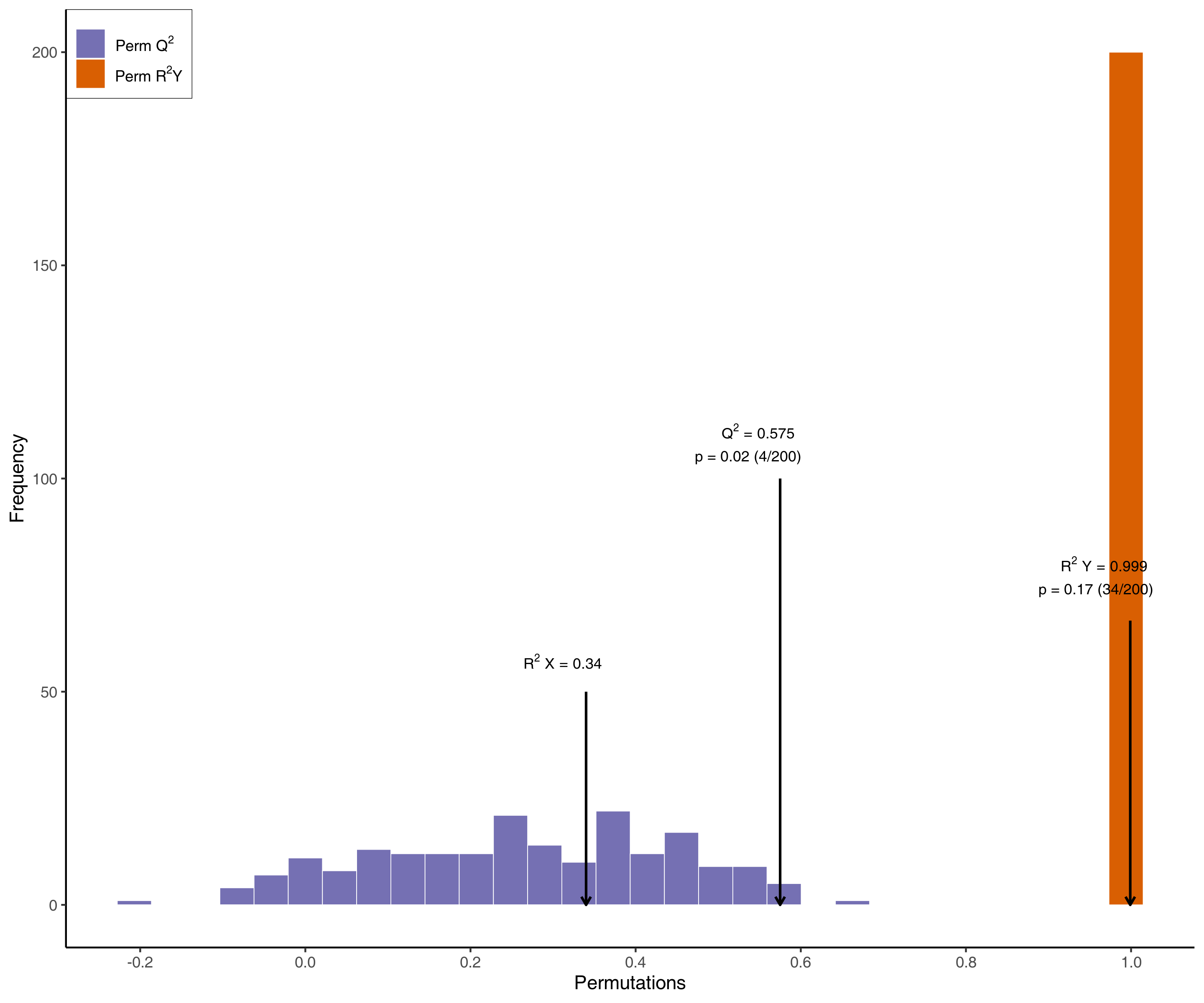

Supplement: Supplementary file 1 [file biology-14-01613-s001.zip › Figure S1.png]

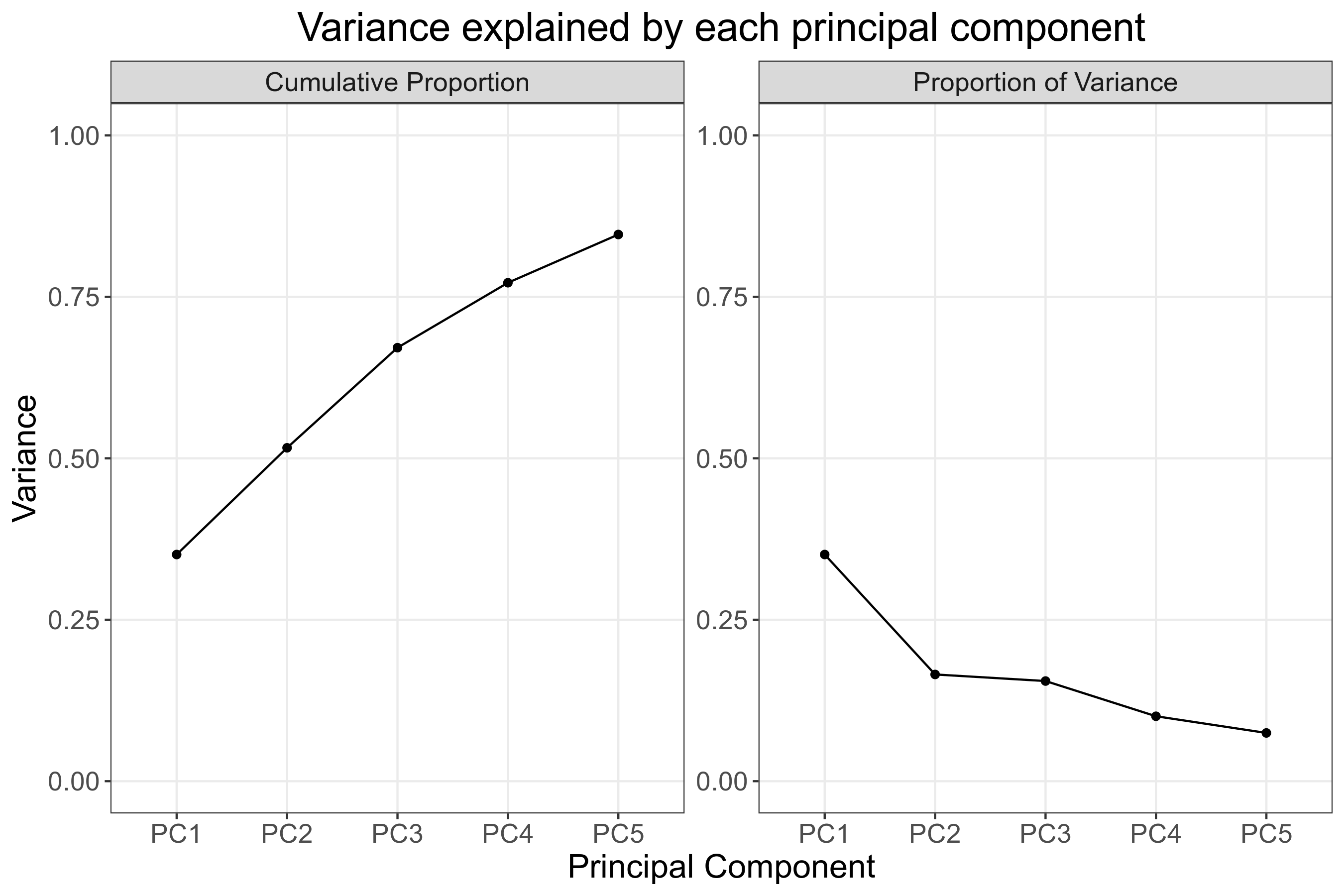

Supplement: Supplementary file 1 [file biology-14-01613-s001.zip › Figure S2.png]

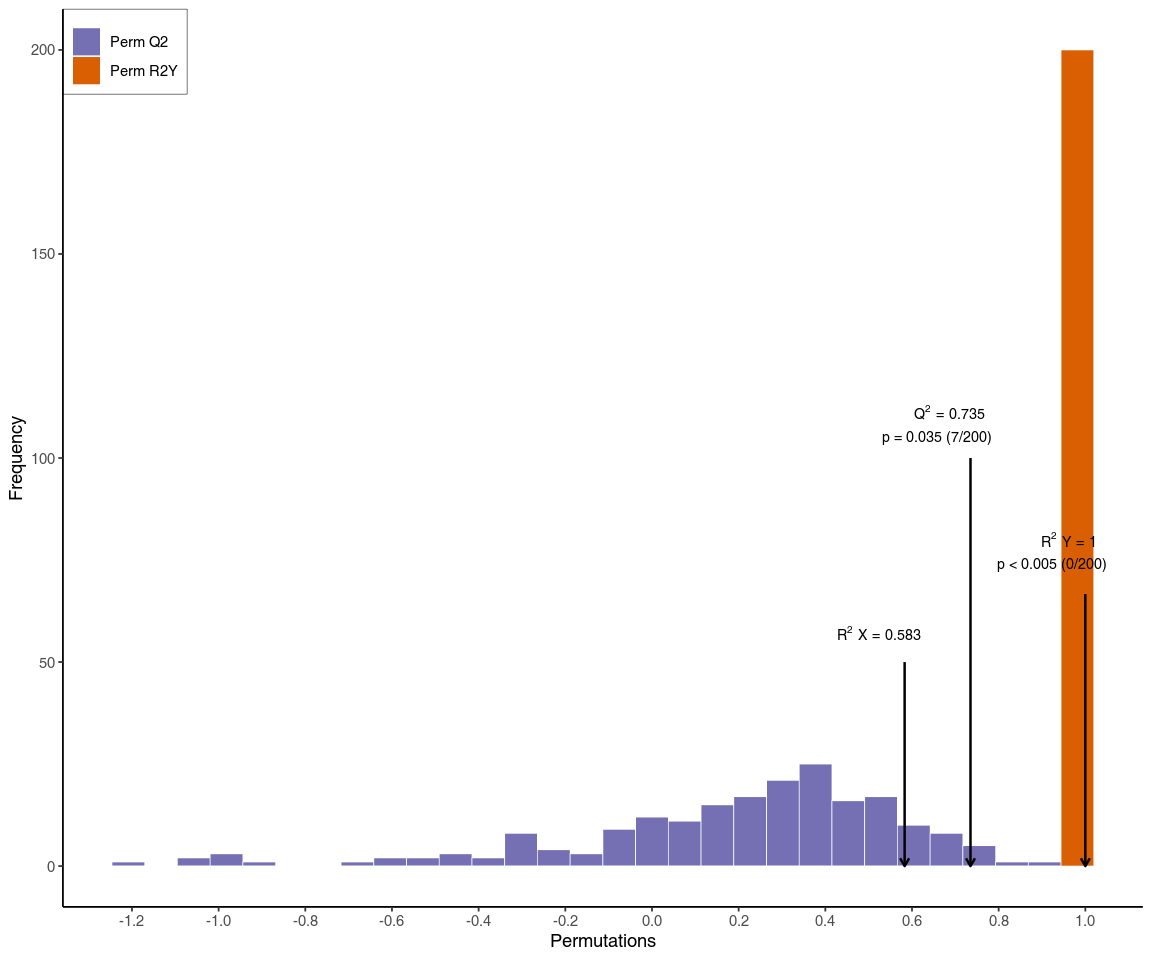

Supplement: Supplementary file 1 [file biology-14-01613-s001.zip › Figure S3.png]

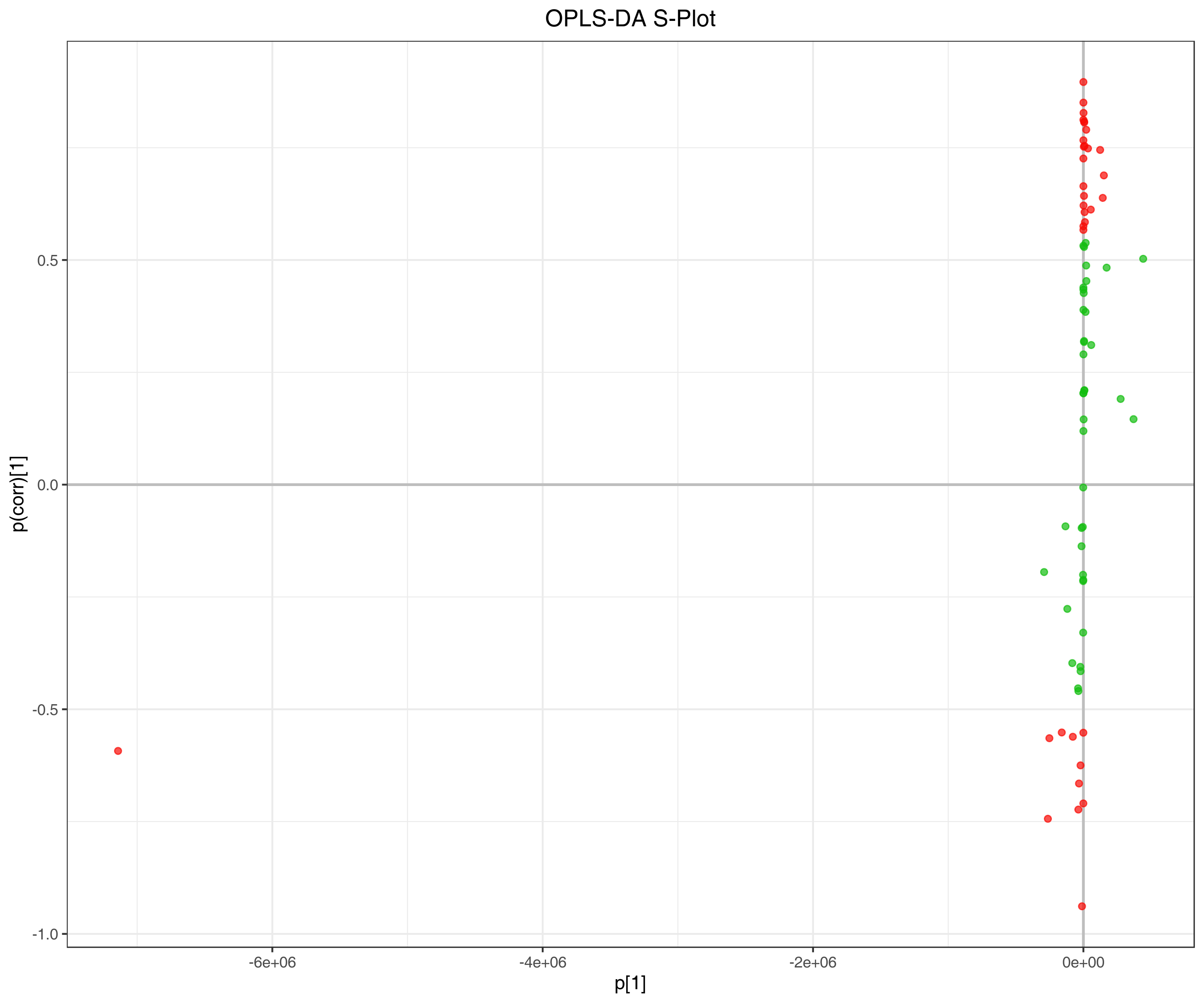

Supplement: Supplementary file 1 [file biology-14-01613-s001.zip › Figure S4.png]

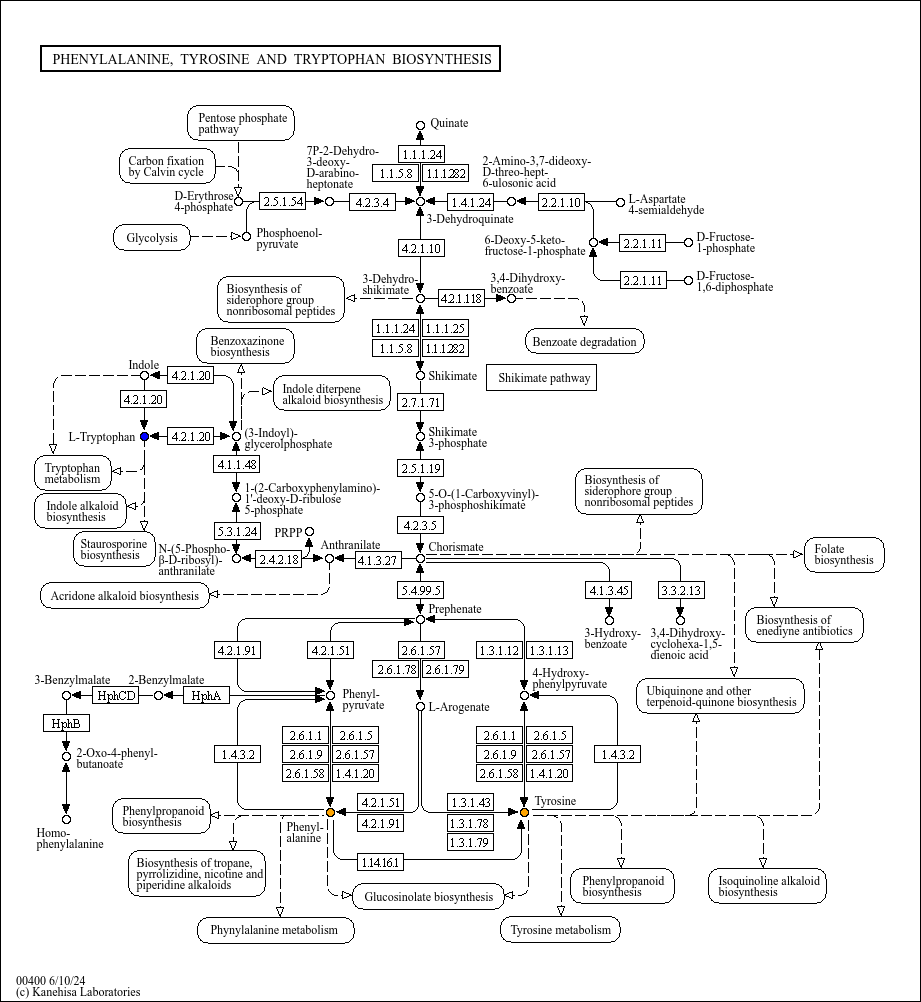

Supplement: Supplementary file 1 [file biology-14-01613-s001.zip › Figure S5.png]

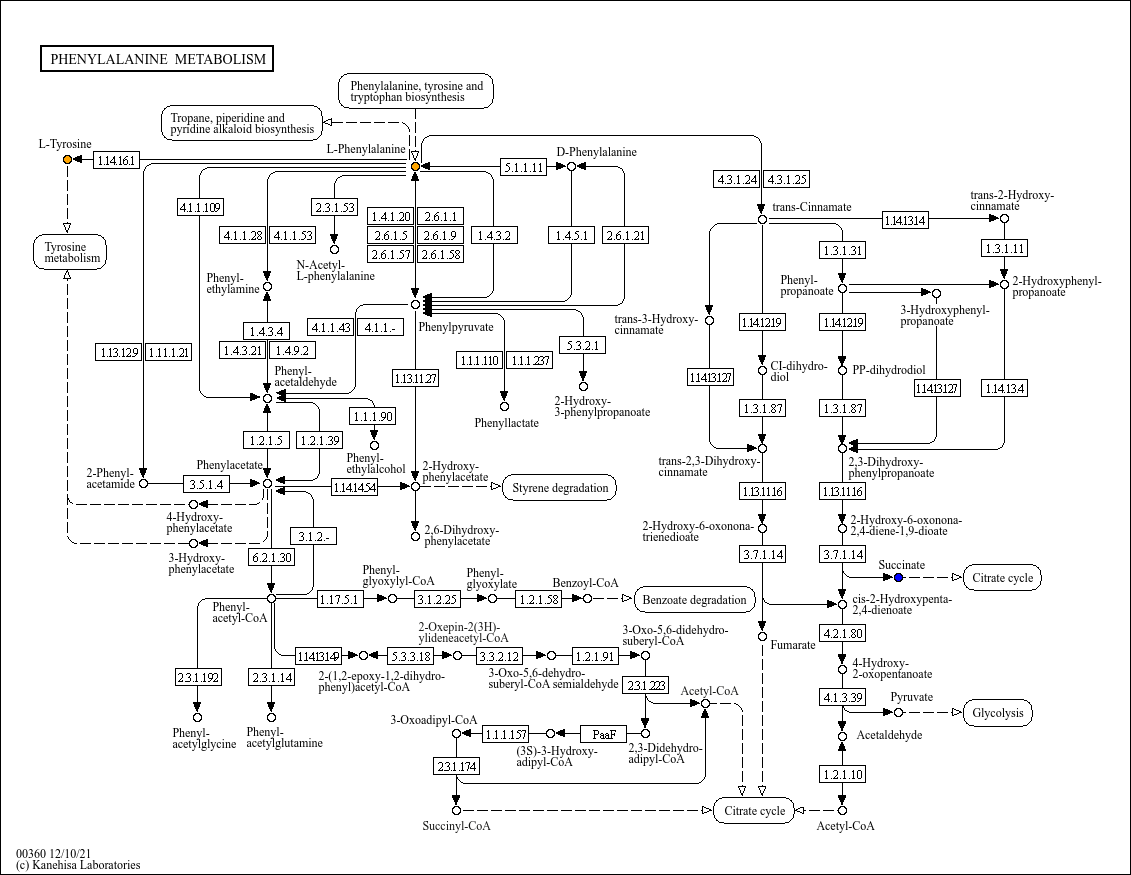

Supplement: Supplementary file 1 [file biology-14-01613-s001.zip › Figure S6.png]
